# *MoB*_2_ Driven Metallic Behavior and Interfacial Charge Transport Mechanism in *MoS*_2_/*MoB*_2_ Heterostructure: A First-Principles Study

**DOI:** 10.1038/s41598-018-32850-z

**Published:** 2018-09-27

**Authors:** Amreen Bano, Devendra K. Pandey, Anchit Modi, N. K. Gaur

**Affiliations:** Department of Physics, Barkatullah University, Bhopal, 462026 India

## Abstract

We have performed the density functional theory calculations on heterostructure (HS) of *MoS*_2_ and *MoB*_2_ monolayers. The aim of this study is to assess the influence of *MoB*_2_ on electron transport of adjacent *MoS*_2_ layer. In present investigation we predict that the electronic properties of *MoS*_2_ monolayer is influenced by 4d-states of Mo in *MoB*_2_ monolayer. Whereas, the B atoms of *MoB*_2_ and S atoms of *MoS*_2_ exhibit overlapping of intermediate atomic orbitals thereby collectively construct the interfacial electronic structure observed to be metallic in nature. From charge density calculations, we have also determine that the charge transfer is taking place at the interface via B-2p and S-3p states. The bonds at the interface are found to be metallic which is also confirmed by adsorption analysis. Thermoelectric performance of this HS is found be in good agreement with available literature. Low Seebeck coefficient and high electrical conductivity further confirms the existence of metallic state of the HS.

## Introduction

The family of layered dichalcogenide semiconductor materials such as molybdenum disulfide (*MoS*_2_) has obtained significant attention over the last years as potential candidates for electronic^1–4^ and optoelectronic applications^5–7^. These materials consist of stacked quasi two dimensional atomic layers that are physically independent to a large extent and can be separated easily. The combination of two-dimensional (2D) dichalcogenides with other 2D or low dimensional materials, such as graphene^7,8^ boron nitride or other dichalcogenides have interesting applications^9^. In principle it provides a completely new class of artificial materials with designed electronic, optical and mechanical properties^10,11^. Tuning of electronic properties of 2D transition metal dichalcogenide (TMD) materials are important for their application in optoelectronics. Monolayer TMDs with formula *MX*_2_ (M = Mo, W; X = S, Se, Te) describes the class of semiconductors with narrow direct band gap, large exciton binding energies, high photoelectrochemical activity and high optoconductivity. Such materials, due to inversion symmetry breaking are used for the study of valley polarization and spin-vally coupling^12,13^. Heterostructures (HSs) are used on a large scale in conventional semiconductors for achieving tunable electronic properties. For the development of future 2D materials, the HSs with Van der Waals interactions have been recognized as promising candidates^14^ and TMD-based hybrid multilayered structures are a prototype van der Waals HSs^15^. It has been reported that the protected phosphorene and tunable carrier dynamics and optical properties can be achieved by coupling of phosphorene with stable graphene^16^ or hBN^17^ and even phosphorene suboxide^18^ and organism *TiL*_4_^19^. According to a theoretical study, phosphorene/graphene HS has been proposed as an anode material for rechargeable Li batteries^20^. The phosphorene/*MoS*_2_ HS is a suitable type-II semiconductor for ultraviolet photodetector applications^21^. Moreover, the black-phosphorus/blue-phosphorus HS is predicted to be used in solar energy conversion^22,23^. Rapid transfer of photogenerated charge carriers between *MoSe*_2_ and graphene has been found in *MoSe*_2_/graphene HSs depicting its applications in optoelectronics^24^; black phosphorene/*MoSe*_2_ HS show potential applications in p-n diodes and logical devices^25,26^. Insulating perovskite substrate *SrTiO*_3_ has also been coupled with *MoS*_2_ monolayer that shows direct semiconducting band gap and chemisorption of *MoS*_2_ monolayer has been observed^27^. Cathodoluminescence and photon emission excited by a high-energy electron beam can be applied in the analysis of mineral compositions^28^, light emitting diodes^29,30^ and surface plasmon mapping^31^. Compared to photoluminescence the cathodoluminescence offers a much higher excitation energy allowing the study of wide band gap materials including diamond^32^ and hexagonal boron nitride (hBN)^33^. The thermoelectric (TE) properties in few layer and bulk *MoS*_2_ and *MoS*_2_ monolayer have been theoretically investigated and found to be reasonably good for TE applications^34–37^. TE power factor of 8.5 *mWm*^−1^ *K*^−2^ for *MoS*_2_ monolayer at room temperature had been reported by Kedar *et al*. which is the highest among all TE materials and twice that of commercially used bismuth telluride (*Bi*_2_*Te*_3_)^38^. Whereas, for *MoS*_2_ monolayer deposited on substrates, Hasan *et al*. had reported a poor response in the TE power factor^39^. For improving the TE performance of *MoS*_2_ based systems, the hybridization and doping have been commonly explored. Moreover SiGe alloys, hybrid BN/graphene and *MoS*_2_/*WS*_2_ nanoribbons show higher thermoelectric properties than single nanostructures^40,41^. We reviewed that the investigation for HSs of *MoS*_2_ with intermetallic material need to be explored. The transition metal diborides *XB*_2_ (X = V, Nb, Ta, Cr, Mo, and W) having hexagonal *AlB*_2_ structure received much attention because for their interesting physical and chemical properties such as electronic structure, high melting point, corrosion resistance, wear resistance, high hardness factor and possibility of extensive industrial applications^16,42–46^. Recently, *ReB*_2_ and *OsB*_2_ has been analyzed for exhibiting high bulk elastic moduli in a particular direction(c-axis) owing to the high valence electron density in the lattice which is comparable to that of diamond^47,48^. Recently it is been reported that lattice mismatches in the cell parameters and comparable thermal expansion with GaN, *ZrB*_2_ can be optimized as a substrate for hetero epitaxial growth of GaN^49,50^. For the reinforcements in various composite materials e.g. steel and *TiB*_2_ has often been used^51,52^. With reference to the above discussion we have motivated to theoretically examine the electronic and thermoelectric properties of *MoS*_2_/*MoB*_2_ HS. We have selected this combination of monolayers due to their interesting electronic properties, specifically the Boron terminated *MoB*_2_ layer with a wide range of thermodynamically allowed chemical potentials^53^. In Section I, we have elaborated the computational details applied to probe several significant features of interfacial electronic structure of *MoS*_2_/*MoB*_2_. The results for electronic structure of HS and isolated sub-systems along with chemisorption, chemical bonding and thermoelectric properties occurring at the interface near to the Fermi level (*E*_*f*_) are reported and discussed in Section II.

## Computational Details

High-throughput density functional theory (DFT)^54^ calculations were performed with the Quantum Espresso simulation package^55^ within the generalized gradient approximation proposed by Perdew, Burke, and Ernzerhof (PBE)^56^. We sampled the Brillouin zone (BZ) in the Monkhorst-Pack scheme^57^, and tested the convergence in energy as a function of number of k-points for the calculations. The k-point sampling of (7 × 7 × 1) was found to be suitable for the BZ corresponding to the primitive unit cell. Atomic positions were optimized using conjugate gradient method, where total energy and atomic forces were minimized. The energy convergence value between two consecutive steps was chosen as 10^4^ eV. The energy interval chosen for density of states (DOS) calculations is 0.1 eV and the broadening used in Gaussian type. The standard value of broadening is considered as 0.001 Ry. An equivalent plane wave cutoff of 750 eV is chosen in all the simulations. Relaxed geometries are obtained with the conjugate gradient method, where all the atoms in the super cell are allowed to relax until the force on each atom is less than 0.02 eV/*Å*. We modeled the *MoS*_2_/*MoB*_2_ HS by putting a 3 × 3 × 1 super cell of *MoS*_2_ monolayer (lattice constant ${a}_{Mo{S}_{2}}$ = 3.12 *Å*)^58^ on top of a 3 × 3 × 1 super cell of *MoB*_2_ monolayer (${a}_{Mo{B}_{2}}$ = 2.98 *Å*)^53^, which reduced the lattice mismatch between the two layers to 4.4% and resulted in a simulation cell containing 32 atoms. This lattice mismatch is small enough that will not effect the electronic properties of the HS, however such contraction in lattice spacings may results in increased DOS^16^. To minimize interactions between periodic images due to 3D boundary conditions, we introduced a vacuum layer such that the distance between periodic images was at least 25 *Å*. We have modeled the interaction of the valence electrons with the pseudo atomic cores of all the atomic species present in our studied structures by normconserving pseudopotentials explicitly including the semi-core Mo 4d electrons in the calculations. The equilibrium interfacial distance (*d*_*eq*_) between *MoS*_2_ and surface of *MoB*_2_ monolayers is found to be 1.96 *Å* which is obtained from a fully relaxed HS. To analyze the thermoelectric properties of HS, semi-classical theory of the Boltzmann package^59^ has been used.

## Results and Discussion

### Electronic Structure

#### *MoS*_2_/*MoB*_2_ Heterostructure

**Figure 1:**
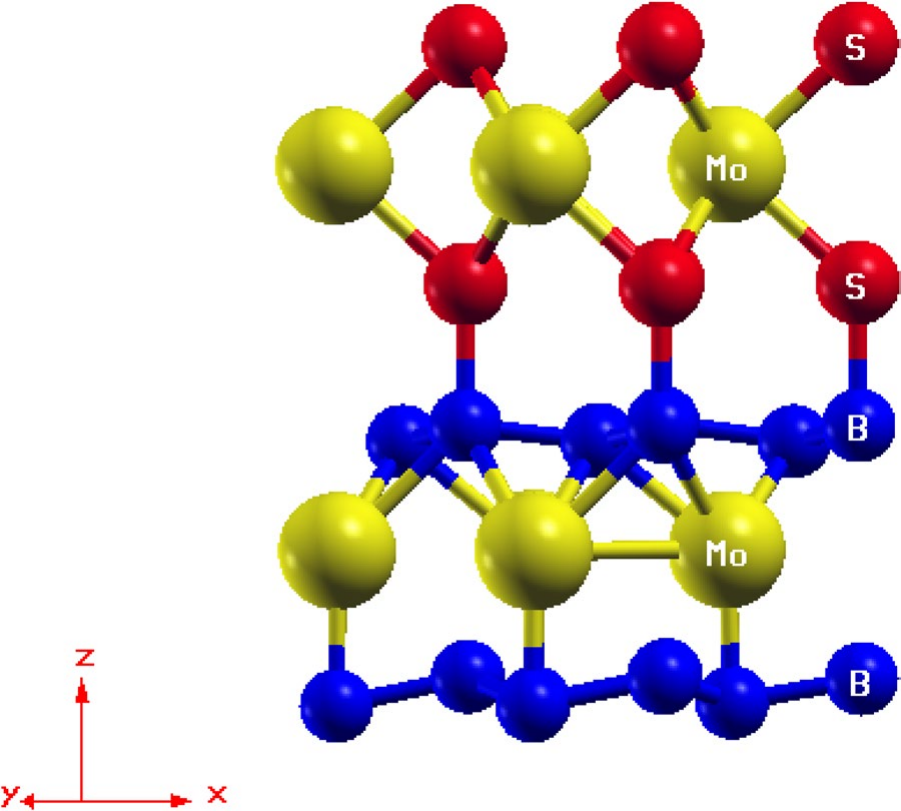
Lattice arrangement of *MoS*_2_/*MoB*_2_ HS. The formation of bonds at the interface is clearly visible.

**Figure 2:**
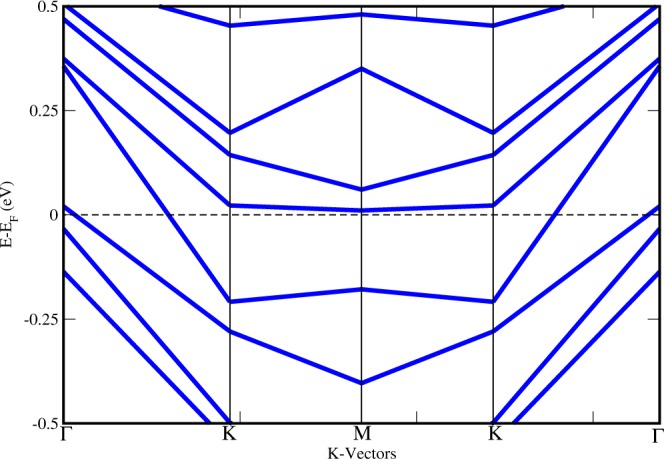
Electronic band structure of *MoS*_2_/*MoB*_2_ HS showing the metallic nature of the HS. The *E*_*F*_ is set to zero reference level.

**Figure 3:**
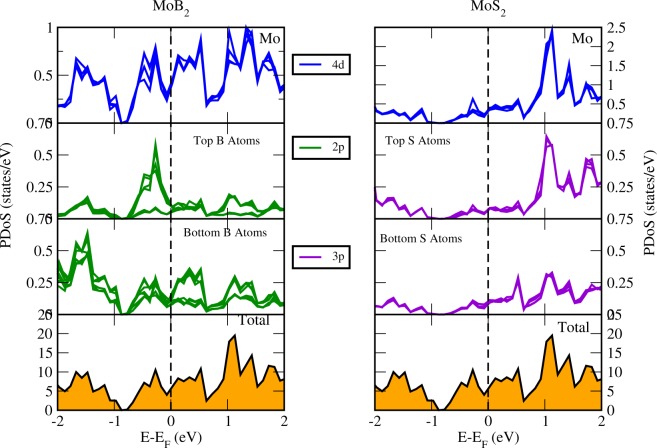
Projected density of states of *MoS*_2_/*MoB*_2_ HS. Left panel shows the contribution of *MoB*_2_ and right panel shows the contribution of *MoS*_2_ in the electronic structure of HS.

The lattice arrangement of the *MoS*_2_/*MoB*_2_ HS shown in Fig. 1 represents the clear existence of bonds at the interface between bottom S atoms and surface B atoms. These bonds indicates that the interaction among the atoms at the interface is not the Van der Waals interaction. In bulk, *MoB*_2_ is a non-layered structure with metallic bonding^16,53^ unlike *MoS*_2_ which possess a layered structure with interlayer Van der Waals interaction. The metallic bonding of *MoB*_2_ is possibly the reason for the absence of Van der Waals interaction at the interface of the HS. The electronic band structure of HS is shown in Fig. 2 where crossing of bands can be seen across the *E*_*F*_ showing the existence of metallic nature. The maximum dispersion of electronic bands is observed at high symmetry point M. In order to further elucidate the band structure we have studied the partial density of states (PDoS) of the HS. Figure 3 is showing the individual contributions of Mo atoms, (top/bottom) B atoms in *MoB*_2_ (left panel) and similarly Mo and (top/bottom) S atoms in *MoS*_2_ (right panel) along with total density of states (DoS) at the bottom. In Fig. 3, PDoS of *MoB*_2_ (left) is shown, where 4d states of Mo atoms are found to cross the *E*_*f*_. The PDoS of B atoms (below Mo) represents the 2p states which are also playing important role in the metallic nature of the HS. Whereas, PDoS of *MoS*_2_ monolayer is represented in Fig. 3 (right). Here we find that, unlike the semiconducting nature of *MoS*_2_ monolayer, 4d states of Mo are observed to cross over the *E*_*f*_. However, the relative intensity of 4d states of Mo in *MoS*_2_ monolayer (0.35 states/eV) is lower than that of 4d states of Mo in *MoB*_2_ monolayer (0.47 states/eV) at *E*_*f*_. 3p states of S atoms (below Mo) are also contributing in the metallic state of HS. The relative intensity of 3p states of S atoms in *MoS*_2_ monolayer (0.11 states/eV) is lower than the 2p states of B atoms in *MoB*_2_ monolayer (0.18 states/eV). From the above results obtained from Fig. 3, we conclude that the major contribution in the metallic character of *MoS*_2_/*MoB*_2_ HS is attributed to Mo-4d and B-2p states of *MoB*_2_ monolayer. *MoB*_2_ is not only making the HS a metallic system but it has also modulated the *MoS*_2_ monolayer to behave as a metallic system. The valance band (VB) in the range from −0.8 to −2 eV, is mainly composed of B-2p and Mo-4d (of *MoB*_2_) states. Whereas, in the conduction band (CB), the energy range from 0.7 to 2 eV is mainly composed of Mo-4d states (of *MoS*_2_ and *MoB*_2_) and S-3p states. From energy range −0.7 to 0.5 eV, dominance of Mo-4d and B-2p states can be clearly seen in Fig. 3. The metallic nature in such type of HSs can be optimized for device applications like gas sensors based on resistivity alterations of the system. Band gaps obtained for other HSs based on *MoS*_2_ are enlisted in Table 1. The PDoS diagrams of the sub-systems of the HS i.e. *MoS*_2_ and *MoB*_2_ are also studied to get a clear insight of the mechanism taking place within the HS and at its interface.

**Table 1:** Band gaps obtained in other works and present work.

Heterostructure	Band Gap *E*_*g*_ (eV)
*MoSe*_2_/*MoS*_2_	0.74^15^
*WS*_2_/*MoS*_2_	1.16^15^
*FeSe*_2_/*MoS*_2_	Metal^15^
*VS*_2_/*MoS*_2_	Metal^15^
*VSe*_2_/*MoS*_2_	Metal^15^
*MoS*_2_/*SrTiO*_3_	0.85^27^
*MoS*_2_/*MoB*_2_	Metal *[This work]*

#### Subsystems: *MoS*_2_ and *MoB*_2_ Monolayers

**Figure 4:**
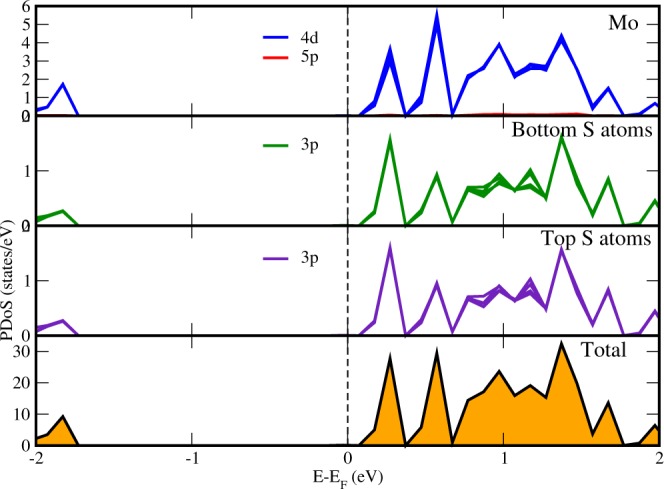
Projected density of states of *MoS*_2_ monolayer on removing the *MoB*_2_ layer from the HS.

**Figure 5:**
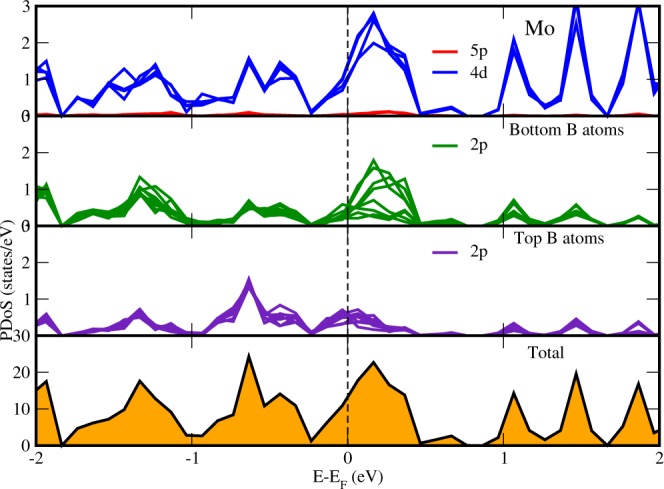
Projected density of states of *MoB*_2_ monolayer on removing the *MoS*_2_ layer from the HS.

To get a clear insight of electron transport, sub-systems (*MoS*_2_ and *MoB*_2_ monolayers) of the HS are also studied separately. The PDoS of *MoS*_2_ monolayer in same lattice arrangement of HS, in absence of *MoB*_2_ monolayer is shown in Fig. 4. We can see here that, when *MoB*_2_ monolayer is not present, *MoS*_2_ is giving an energy gap of 1.8 eV as expected from it^60^. 4d states of Mo and 3p states of S atoms are dominantly found in the CB near *E*_*f*_ level. The relative intensity of peaks of 4d states of Mo atoms (3.8 states/eV) are higher than that of 3p states of S atoms (1.6 states/eV). The PDoS of lower S atoms and top S atoms are identical. In the VB, there is a vacant space upto −1.7 eV which shows that the electronic states of *MoS*_2_ monolayer are present in the VB in lower energy region ≤−1.7 eV. On observing Fig. 4, it is clear that, *MoB*_2_ has strongly influenced the electronic states of *MoS*_2_ monolayer when coupled together in the HS *MoS*_2_/*MoB*_2_ making it a metal. The PDoS of *MoB*_2_ monolayer when *MoS*_2_ monolayer is removed from the HS, is shown in Fig. 5. We can see here that 4d states of Mo are crossing the *E*_*f*_ level making it a metallic material. Below Mo, PDoS of bottom and top B atoms are shown. We found in Fig. 5 that 2p states of bottom B atoms are having higher intensity peaks at *E*_*f*_ as compared to the top B atoms, which concludes that 2p states bottom B atoms are more actively participating with 4d states of Mo in making the system *MoB*_2_ a metal. Our findings from Fig. 5 indicates that the interfacial bonds between *MoS*_2_ and *MoB*_2_ monolayers are metallic bonds.

### Chemical Bonding

**Figure 6:**
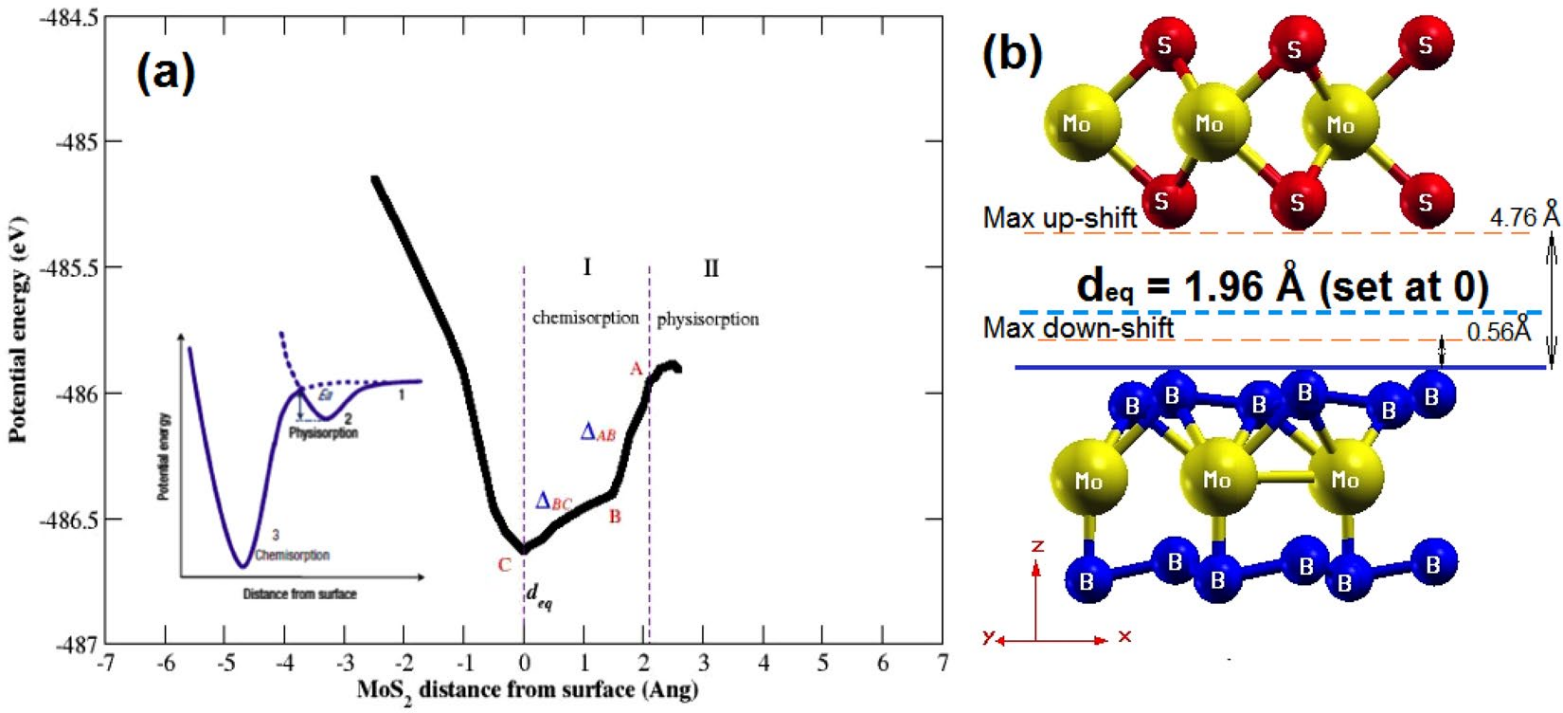
Nature of adsorption exists in the *MoS*_2_/*MoB*_2_ HS. Inset figure shows the ideal curve of adsorption.

**Figure 7:**
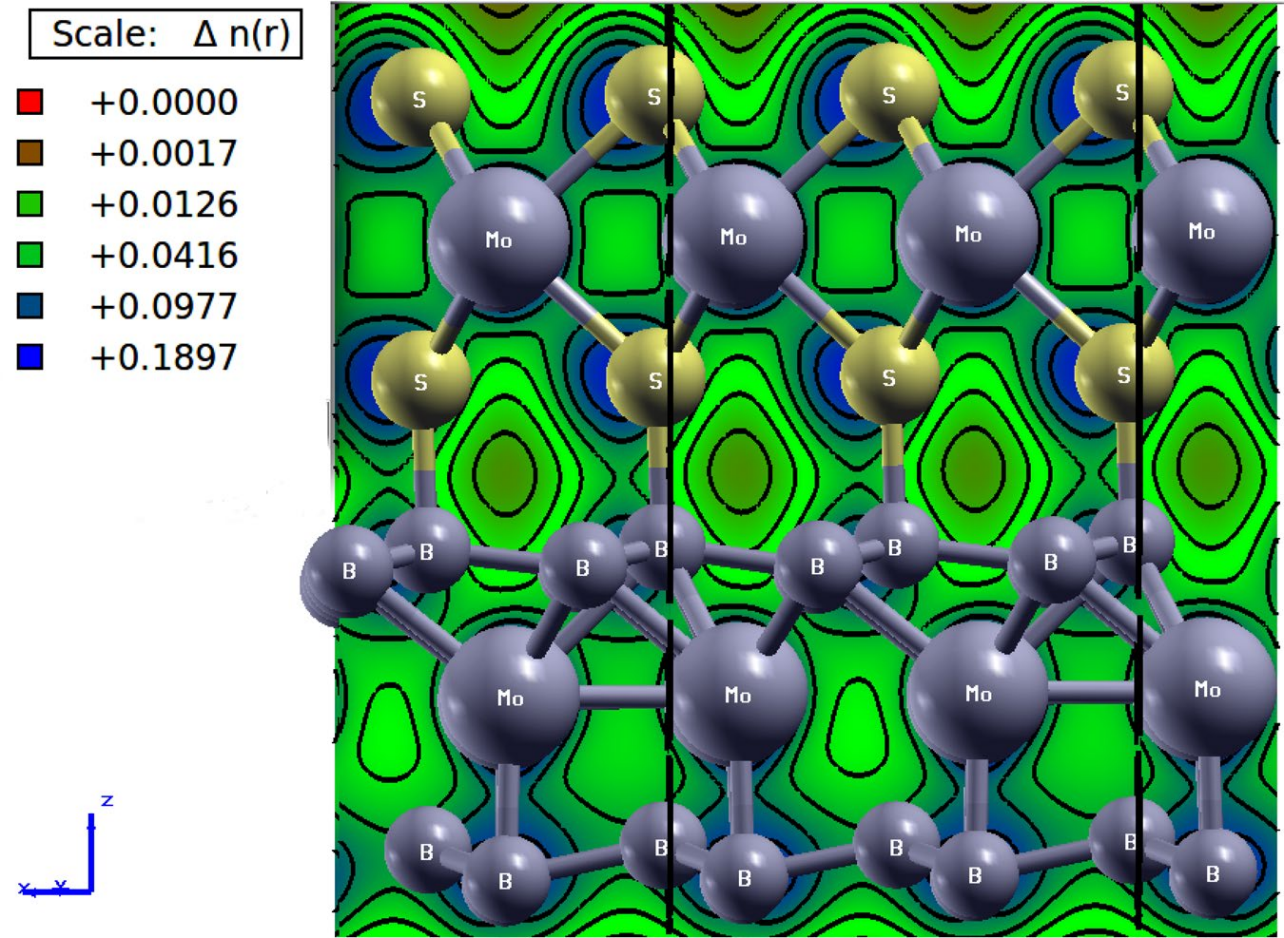
Charge density plot of *MoS*_2_/*MoB*_2_ HS in [010] plane.

**Figure 8:**
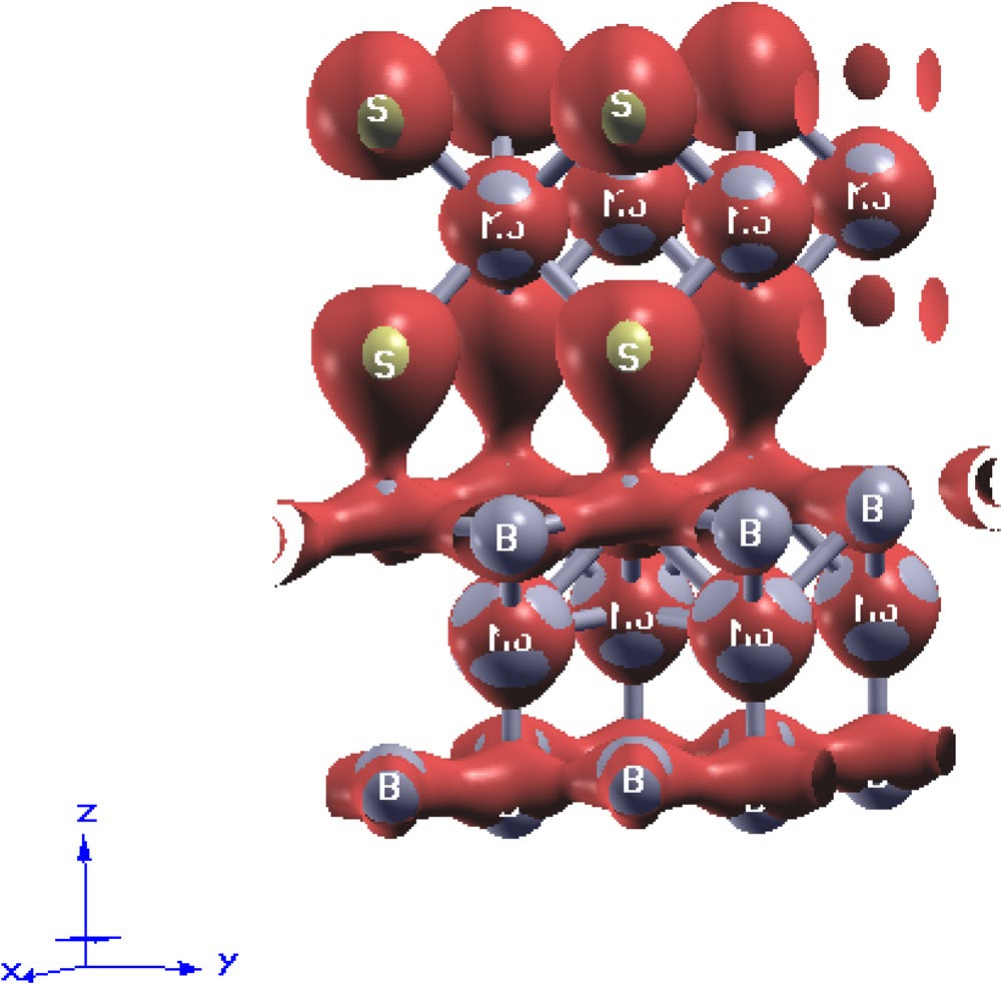
Isosurface results of *MoS*_2_/*MoB*_2_ HS showing the interfacial charge transfer between B-2p and S-3p orbitals.

In the system of present investigation, we have performed the adsorption analysis of *MoS*_2_ deposited on *MoB*_2_ to confirm the metallic nature of interfacial bonding. In chemisorption the chemical reaction occurs between the surface of adsorbent (*MoB*_2_) and adsorbate (*MoS*_2_) and hence, new chemical bonds are generated at the interface along with consequential alterations in the electronic structure of adsorbate. On the other hand, in case of physisorption, the electronic properties of adsorbate remains unaltered upon adsorption. The bonds can be seen at the interface between adsorbent (*MoB*_2_) and adsorbate (*MoS*_2_) in Fig. 1. This *MoB*_2_ driven modulation in the electronic structure of *MoS*_2_ motivated us to investigate the nature of adsorption in *MoS*_2_/*MoB*_2_ HS system. In general, layered *MoS*_2_ exhibits honeycomb like structure at the interface with Van der Waals like weak interactions. In our case we observed the existence of some ionic type bonding between B-2p electronic states of *MoB*_2_ and S-3p electronic states of *MoS*_2_ monolayers. Therefore it is considered that these interfacial interactions may not be the Van der Waals interactions^27^. In order to analyze the nature of adsorption at *MoS*_2_/*MoB*_2_ interface with equilibrium interfacial distance (*d*_*eq*_ = 1.96 *Å*), we have randomly shifted *MoS*_2_ monolayer upward upto 4.76 *Å* and then downward upto 0.56 *Å* normal to the plane of *MoS*_2_/*MoB*_2_ HS. The result obtained from adsorption curve in terms of change in potential energy with respect to interfacial distance is shown in Fig. 6(a), whereas the inset of Fig. 6(a) represents conventional potential energy versus interfacial distance curve. The diagrammatic representation of shifting of *MoS*_2_ monolayer over *MoB*_2_ is provided in Fig. 6(b). With reference to Fig. 6(b) the distance *d*_*eq*_ is set to 0 *Å* which indicates the minima of potential energy curve in Fig. 6(a). Again from Fig. 6(b) the maximum separation between *MoS*_2_ and *MoB*_2_ is ~4.76 *Å* which is then reduced to a minimum value ~0.56 *Å*. In Fig. 6(a) the two regions I and II can be allocated as chemisorption and physisorption respectively. With reference to *d*_*eq*_, the chemisorption occurs in the region from A (maxima at 2.1 *Å*) to C (minima at *d*_*eq*_ = 1.96 *Å*), where chemical bonds possibly exist at the interface of *MoS*_2_/*MoB*_2_ HS. The potential energy curve attains the minimum potential energy value (at C) by change in slope via point B. The rate of change of potential energy with respect to the interfacial distance between A and B i.e. Δ_*AB*_ is greater than that between B and C (Δ_*BC*_). This variation in slope is attributed to initial electronic repulsion. In other words the anomaly at B in the potential energy curve appears due to electronic repulsion between *MoS*_2_ and *MoB*_2_ layers. Tending from B towards C the actual orbital overlapping between the atomic species of corresponding layers can be realized. Conclusively, at C in the potential energy curve the valley-like feature at *d*_*eq*_ indicates a clear existence of chemical bonding at the interface. If we further continue to decrease this distance, the energy will tend to infinity under the effect of nuclear repulsion. We can see from Fig. 6(a), that the trend of adsorption curve represents clear existence of chemical bonds at the interface which justify our results of electronic structure of HS and also confirms that the interfacial bonds are not merely the state-of-art. Now to show the nature of chemical bonding i.e. whether these bonds are metallic or covalent, we have further studied the charge density plot at the interface as shown in Fig. 7. Maximum value in the color plate on left shows the charge accumulation. Moreover the charge transfer occurs between top B atoms (of *MoB*_2_ surface) and bottom S atoms (of *MoS*_2_). This indicates the presence of strong bonding with metallic nature at the interface. Hybridization in Mo-4d states and B-2p states in the *MoB*_2_ monolayer is also observed. In *MoS*_2_ monolayer, Mo atoms weakly participate in charge transfer. These results are in good agreement with those observed for PDoS (Fig. 3) of *MoS*_2_/*MoB*_2_ HS. The isosurface plot of HS in Fig. 8 further elucidates the results of charge density. The interfacial bonding is clearly visible along with the bottom B atoms of the *MoB*_2_ monolayer forming covalent B-B bonds among themselves^16^ and surfacial B atoms (*MoB*_2_ monolayer) making bonds with S atoms as well as B-B bonds.

### Thermoelectric Properties

The performance of a thermoelectric material reflects in the dimensionless figure of merit ZT = *S*^2^*σT*/*κ*, where S is the Seebeck coefficient, *σ* is the electrical conductivity and *κ* (*κ* = *κ*_*e*_ + *κ*_*l*_) is the thermal conductivity which consist electronic *κ*_*e*_ as well as lattice *κ*_*l*_ thermal conductivity and T is absolute temperature respectively. Previous studies signified that the mono-layered *MoS*_2_ is semiconductor in nature with band gap 1.8 eV^60^. Due to high S and low *κ*, *MoS*_2_ system presents a good candidature for the thermoelectric applications. However, small ZT is reported for this system due to low electrical conductivity induced by the large band gap energy. The mono-layered *MoB*_2_ have metallic nature as represented in PDoS (Fig. 5) discussed in above section. We propose that wide band gap of *MoS*_2_ semiconductor can be tuned by HS with *MoB*_2_ that possibly results in enhanced power factor and thereby improve thermoelectric properties. Aiming this, we are the first to attempt the calculation and analysis of thermoelectric properties of *MoS*_2_/*MoB*_2_ HS using BoltzTrap code^59^.

**Figure 9:**
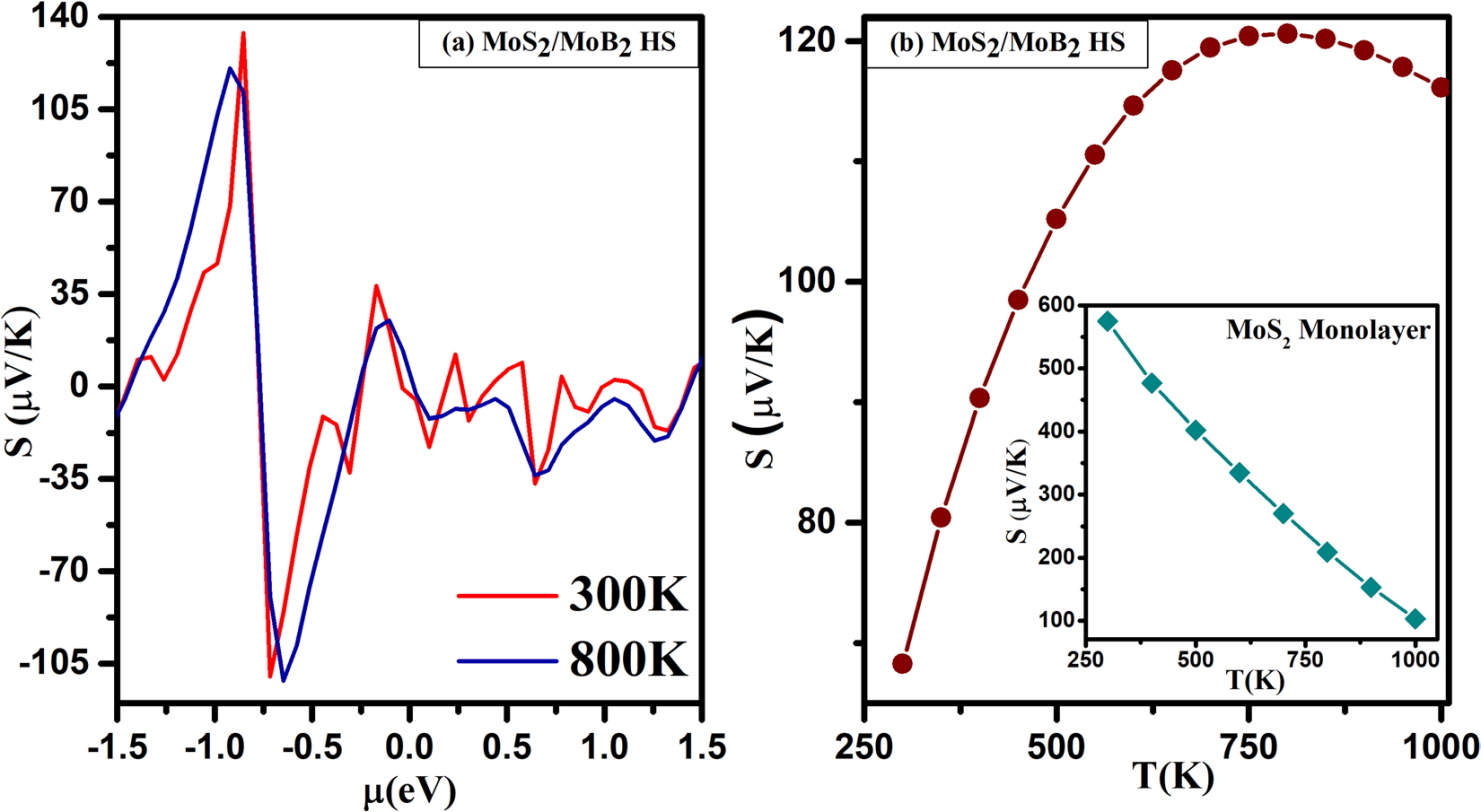
Seebeck coefficient (in *μ*V/K, where *μ* = 10^−6^) varying as a function of (**a**) chemical potential *μ* (in eV) and (**b**) Temperature T(K) of *MoS*_2_/*MoB*_2_ HS and inset of (**b**) Temperature T(K) of *MoS*_2_ Monolayer.

The Seebeck coefficient (S) as a function of chemical potential (*μ*) from −1.5 eV to 1.5 eV at temperatures 300 K and 800 K for *MoS*_2_/*MoB*_2_ HS show two peaks in the profile (Fig. 9a) which are located at a chemical potential near around −0.85 eV and −0.91 eV. It can be noticed that the resultant magnitude is larger for a p-type character. The maximum value of S is 134 *μ*V/K, at 300 K which decreases with increasing temperature. The perpendicular component is higher in magnitude which is good for thermoelectric properties. The temperature dependence of Seebeck coefficient at a certain value of chemical potential is shown in Fig. 9(b). For *MoS*_2_/*MoB*_2_ HS the values of S in the entire temperature range are found to be positive which reveals that p-type charge carriers are dominant and increases with increasing temperature. Dimple *et al*.^41^ observed the thermoelectric properties of *MoS*_2_ monolayer which signify p-type character. However, the magnitude of S is very low (=10^−6^) due to metallic nature of *MoS*_2_/*MoB*_2_ HS. The *MoS*_2_ monolayer is a semiconductor that possess large band gap therefore its Seebeck coefficient must be larger than that of purely metallic *MoB*_2_ which is shown in inset of Fig. 9(b). Moreover with reference to the electronic structure as discussed above for this HS, the band gap of *MoS*_2_ monolayer is modulated by *MoB*_2_. It can also be observed from PDoS of HS (Fig. 3) that the band gap of HS acquires metallic behavior and hence the Seebeck coefficient of mixed-layer *MoS*_2_/*MoB*_2_ is smaller than *MoS*_2_ monolayer^61^.

**Figure 10:**
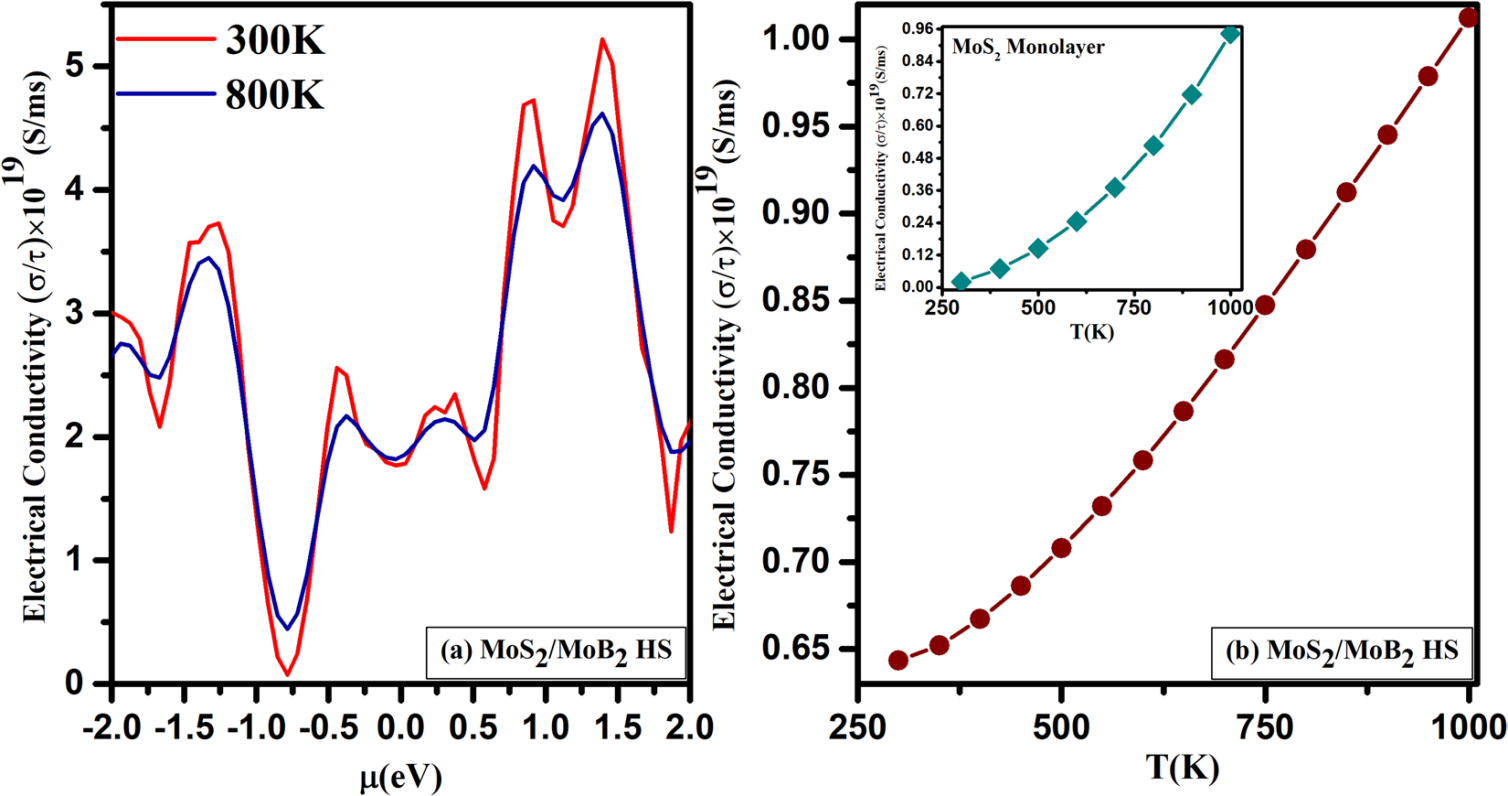
Electrical conductivity as a function of (**a**) chemical potential (*μ*(*eV*)) and (**b**) Temperature T(K) of *MoS*_2_/*MoB*_2_ HS and inset of (**b**) Temperature T(K) of *MoS*_2_ Monolayer.

The variation of electrical conductivity (*σ*/*τ*) as a function of chemical potential (*μ*) from −2 eV to 2 eV at the temperatures 300 K and 800 K for *MoS*_2_/*MoB*_2_ HS is shown in Fig. 10(a). We have observed that the electrical conductivity for negative chemical potential is 3.73 × 10^19^ (*S*/*ms*) and 3.423 × 10^19^ (*S*/*ms*), whereas for positive chemical potential it is 5.21 × 10^19^ (*S*/*ms*) and 4.588 × 10^19^ (*S*/*ms*) for 300 K and 800 K respectively. This indicates that the p-type composition possess higher electrical conductivity than n-type. Comparable phenomena had also been observed in *MoS*_2_ monolayer^41^. Further, the electrical conductivity (*σ*/*τ*) as a function of temperature for a certain value of chemical potential (*μ*) is shown in Fig. 10(b). This figure shows that the electrical conductivity increases almost linearly with increasing temperature which indicates metallic nature, also confirmed by DoS study (Fig. 3). The electrical conductivity of *MoS*_2_ monolayer have been provided in the inset of Fig. 10(b) which shows a similar trend like *MoS*_2_/*MoB*_2_ HS. However, the electrical conductivity of *MoS*_2_ monolayer found lesser as compared to the investigated HS.

**Figure 11:**
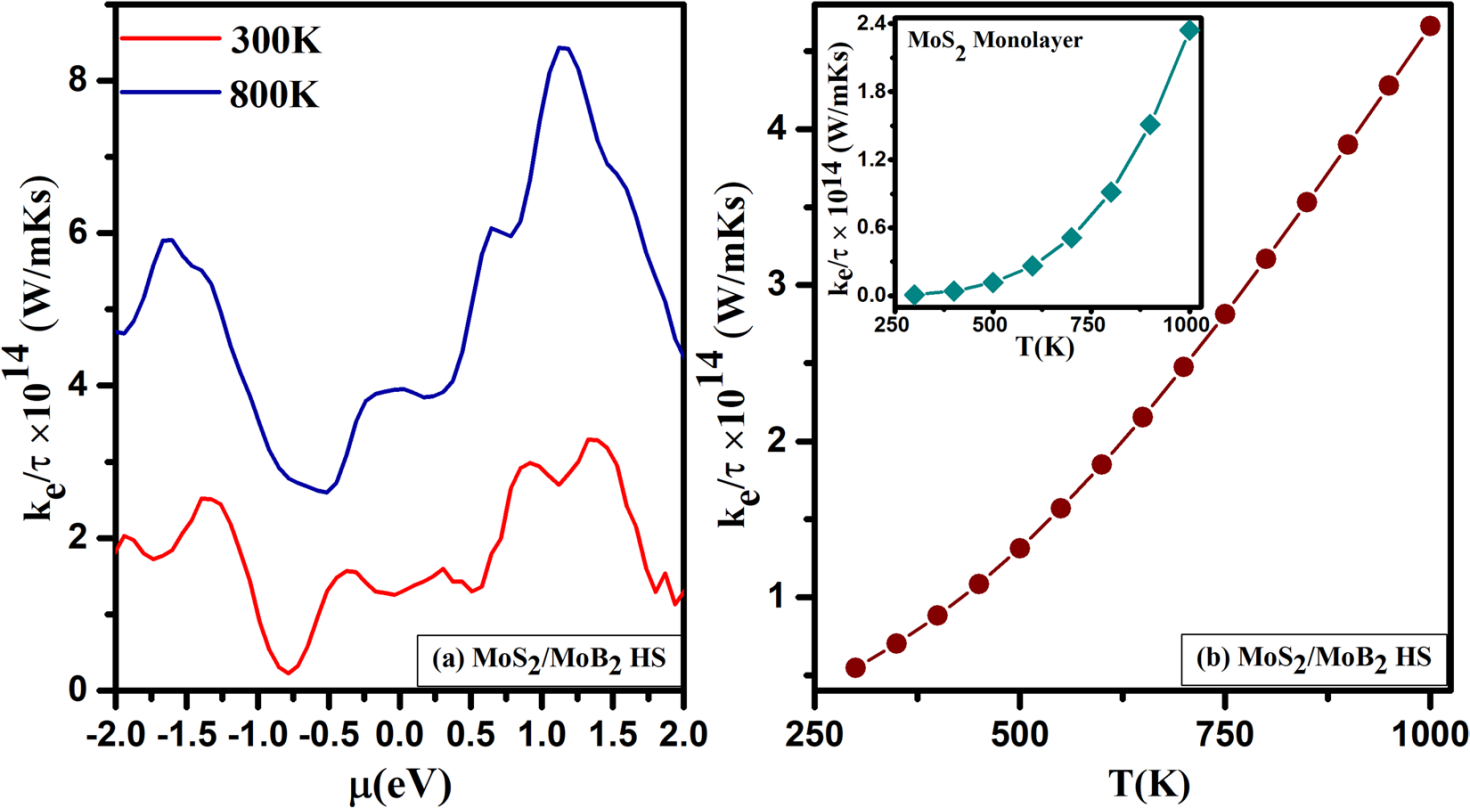
Thermal conductivity as a function of (**a**) chemical potential (*μ*(*eV*)) and (**b**) Temperature T(K) of *MoS*_2_/*MoB*_2_ HS and inset of (**b**) Temperature T(K) of *MoS*_2_ Monolayer.

Further, we have also focused on the thermal conductivity of *MoS*_2_/*MoB*_2_ HS. In general, the thermal conductivity *κ* (*κ* = *κ*_*e*_ + *κ*_*l*_) is a combination of electronic thermal conductivity *κ*_*e*_ and phononic thermal conductivity *κ*_*l*_. In the present study, we have used the BoltzTrap code^59^ which calculates electronic contribution only. The consideration of lattice thermal conductivity *κ*_*l*_, remains as future task. The calculated electronic thermal conductivity (*κ*_*e*_/*τ*) of *MoS*_2_/*MoB*_2_ HS as a function of chemical potential (*μ*) at 300 K and 800 K is shown in Fig. 11(a). It is indicated that a significant increase in *κ*_*e*_/*τ* occurs with increasing temperature. The highest value of *κ*_*e*_/*τ* induced by 800 K while the lowest *κ*_*e*_/*τ* induced by 300 K. Therefore, 300 K is the optimal temperature that gives the lowest thermal conductivity. Moreover, the variation in thermal conductivity with respect to temperature is represented in Fig. 11(b). It shows a linear dependence with respect to temperature because the increasing temperature enhances the number of charge carriers attributed to the metallic nature of *MoS*_2_/*MoB*_2_ HS which is in good agreement with the previous study^62^. However, the thermal conductivity of *MoS*_2_ is found less as compared to that of *MoS*_2_/*MoB*_2_ HS as shown in the inset of Fig. 11(b). It is due to the large band gap semiconducting nature of *MoS*_2_ monolayer^63^.

**Figure 12:**
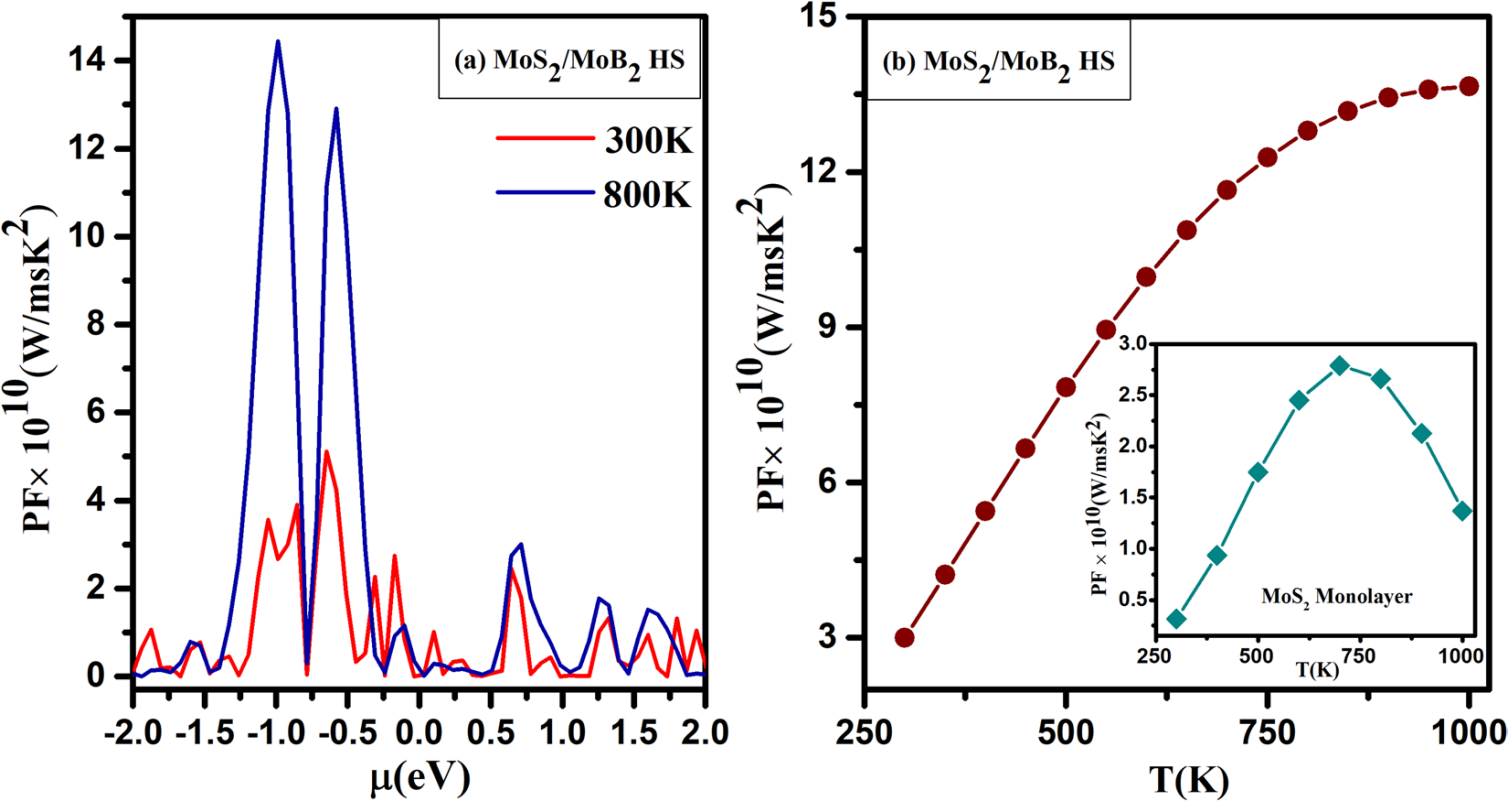
Power factor as a function of (**a**) chemical potential (*μ*(*eV*)) and (**b**) Temperature T(K) of *MoS*_2_/*MoB*_2_ HS and inset of (**b**) Temperature T(K) of *MoS*_2_ Monolayer.

The average power factor is plotted against the chemical potential (*μ*) at 300 K and 800 K illustrated in Fig. 12(a). The positive (and negative) chemical potential scale indicates the electron (and hole) concentration, respectively. The power factor is maximum near *μ* = −0.99 eV attributed to significant increment in the electrical conductivity at high electron concentration level. Further, Fig. 12(b) represents the power factor with respect to increasing temperature which initially increases rapidly and then becomes almost linear till 1000 K. The comparative power factor of *MoS*_2_ monolayer is shown in the inset of Fig. 12(b). It is clear that the investigated *MoS*_2_/*MoB*_2_ HS exhibits comparatively better power factor value than *MoS*_2_ monolayer. The large magnitude of power factor is obtained in the case of large electrical conductivity. Consequently, it is clear that the heterostructure of *MoS*_2_/*MoB*_2_ HS exhibit good thermoelectric response at the higher temperatures.

## Conclusions

In conclusion, our study suggests that deposition of *MoS*_2_ monolayer over single layer of *MoB*_2_ in a bilayer *MoS*_2_/*MoB*_2_ heterostructure can have noticeable effects on the electronic properties of the *MoS*_2_ layer. In presence of *MoB*_2_ layer in the HS, monolayer of *MoS*_2_ becomes a metallic system. Under the influence of 4d and 2p states of Mo and B atoms respectively of *MoB*_2_ layer, 4d and 3p states of Mo and S atoms of *MoS*_2_ monolayer appeared to cross the *E*_*f*_. While in absence of *MoB*_2_ layer, *MoS*_2_ monolayer shows its ideal electronic structure. Hence the metallic nature of the HS is driven by *MoB*_2_ layer. We also observed some bonds at the interface which were analyzed via charge density calculation and adsorption curve and found to be metallic in nature. Based on the calculated Seebeck effect and power factor of *MoS*_2_/*MoB*_2_ HS as a function of chemical potential and temperature, the maximum power factor is estimated successfully which can offer useful guidelines for tuning and improving the thermoelectric performance of such type of HS.
